# HIV-Care Outcome in Saudi Arabia; a Longitudinal Cohort

**DOI:** 10.4172/2155-6113.1000370

**Published:** 2014-11-08

**Authors:** Maha A. Al-Mozaini, Michael K. Mansour, Abdullah A. Al-Hokail, Magid A. Mohmed, Munirah A. Bin Daham, Hail M. Al-Abdely, Husn H. Frayha, Fahad A. Al-Rabiah, Sami H. Alhajjar, Salmaan Keshavjee, Chaker N. Adra, Abdulrahman A. Alrajhi

**Affiliations:** 1 Immunocompromised Host Research, Department of Infection and Immunity, King Faisal Specialist Hospital and Research Centre, Riyadh, Kingdom of Saudi Arabia; 2 Division of Infectious Diseases, Department of Medicine, Massachusetts General Hospital, Boston, MA, USA; 3 Department of Medicine, King Faisal Specialist Hospital and Research Centre, Riyadh, Kingdom of Saudi Arabia; 4 Department of Global Health and Social Medicine, Harvard Medical School, Boston, MA, USA

**Keywords:** HIV, Human immunodeficiency Virus-1, CD4+T cells, Antiviral therapy, Viral load, Opportunistic infections, AIDS, Acquired immunodeficiency syndrome

## Abstract

**Background:**

Clinical characteristics of HIV-1 infection in people inhabiting Western, Sub-Saharan African, and South-East Asian countries are well recognized. However, very little information is available with regard to HIV-1 infection and treatment outcome in MENA countries including the Gulf Cooperation Council (GCC) states.

**Methods:**

Clinical, demographic and epidemiologic characteristics of 602 HIV-1 infected patients followed in the adult Infectious Diseases Clinic of King Faisal Specialist Hospital and Research Centre, in Riyadh, Kingdom of Saudi Arabia a tertiary referral center were longitudinally collected from 1989 to 2010.

**Results:**

Of the 602 HIV-1 infected patients in this observation period, 70% were male. The major mode of HIV-1 transmission was heterosexual contact (55%). At diagnosis, opportunistic infections were found in 49% of patients, most commonly being pneumocysitis. AIDS associated neoplasia was also noted in 6% of patients. A hundred and forty-seven patients (24%) died from the cohort by the end of the observation period. The mortality rate peaked in 1992 at 90 deaths per 1000 person-year, whereas the mortality rate gradually decreased to <1% from 1993-2010. In 2010, 71% of the patients were receiving highly active retroviral therapy.

**Conclusions:**

These data describe the clinical characteristic of HIV-1-infected patients at a major tertiary referral hospital in KSA over a 20-year period. Initiation of antiretroviral therapy resulted in a significant reduction in both morbidity and mortality. Future studies are needed in the design and implementation of targeted treatment and prevention strategies for HIV-1 infection in KSA.

## Introduction

Human immunodeficiency virus (HIV) infection represents one of the preeminent global health problems and a major health delivery challenge. According to the World Health Organization (WHO) and the joint United Nations program on HIV/AIDS (UNAIDS), 33 million people worldwide are currently infected with HIV (http://www.unaids.org). The use of antiretroviral therapy has dramatically improved the outcome and survival of HIV-infected persons.

A number of analyses of treatment outcomes in longitudinal cohorts from both well-resourced and resource-limited settings have been published [1-9]. However, data on HIV treatment outcomes in the MENA region are limited. In the decade ending in 2009, approximately 24,000 out of the estimated 460,000 people with HIV in the MENA region died of AIDS. Although estimates of the prevalence of the disease remain low (approximately 0.1%-0.2% in Egypt, Iran, Jordan, Libya, Morocco, Syria, Tunisia, Gulf Cooperation Council (GCC) countries, and Yemen), the number of new infections in the MENA region is increasing [10].

HIV is increasingly recognized as a major health problem in Kingdom of Saudi Arabia (KSA) and in the rest of member states in GCC [11]. The first official case of HIV infection in KSA was reported in 1984 in a blood transfusion recipient [12]. By 1986, 13 HIV-infected patients in KSA had been documented, all linked to contaminated blood products [13]. Between 1984 and 2001, 6,046 HIV infections were reported in KSA, 1,285 (21%) of whom were Saudi citizens [14, 15]. In response to the epidemic, a national AIDS program was established within the Saudi Ministry of Health (MOH) to oversee and coordinate country-wide efforts to prevent, diagnose and treat HIV. One of the first care provision facilities was the King Faisal Specialist Hospital and Research Centre (KFSHRC), where the first case had been reported. HIV-infected patients from all provinces of KSA are referred to this institution for counselling, diagnostic evaluation, and treatment. Currently, one out of four HIV-infected patients in KSA is receiving care in this facility. Clinical and demographic data from these patients have been entered longitudinally into a central database. This database is primary data source for HIV treatment outcomes in Saudi Arabia. The purpose of this study is to describe HIV treatment outcomes in a cohort of 602 prospectively enrolled, longitudinally-followed HIV patients, whom all are Saudi National from different social- economic background.

## Methods and Materials

### Patient Cohort

HIV-infected patients were collected in this observational cohort from 1989 to 2010 at the KFSHRC. Over the 20-year period, 602 patients all of whom are Saudi Nationals were enrolled representing 1,200 annual patient-visits. Broadly speaking, patients in the cohort belonged to three groups: 1) newly diagnosed HIV patients within the hospital’s catchment area (around 5 new cases per year, mostly contacts of recently diagnosed patients-spouses, parents or children); 2) referral from the Ministry of Health (MOH) or other care providers; and 3) self-referrals. The data were entered into a customized database that consisted of 150 variables of patient information; including demographic data such as geographic origin, age, and gender. In addition, patient’s medical information was collected by chart review to include age at presentation, mode of transmission, symptoms, laboratory analyses, antiretroviral therapy, and opportunistic infections.

### HIV diagnostics

Until 1996, HIV diagnosis was established by enzyme immunoassay with confirmation by immunoblot assay. After 1997, HIV polymerase chain reaction (PCR) was used to confirm the diagnosis in patients with reactive serologic tests.

Initial serologic testing for HIV 1 and 2 was performed using a micro-particle enzyme immunoassay (MEIA; AxSYM HIV 1/2 gO, Abbott Laboratories, Abbott Park, USA). If serology was reactive, specimens were confirmed using the Chiron RIBA HIV-1/HIV-2 strip immunoblot assay.

For the purpose of this report, we used the CDC definition of AIDS [16]. Opportunistic infections are defined by microbiological confirmation (by stain for Pneumocystis jirovecii, culture for M. tuberculosis, atypical mycobacteria, and fungi).

Molecular testing included HIV PCR, which uses three sets of four primers from envelope, polymerase, reverse transcriptase, and core protein genes. Viral load was measured with the branched DNA method (VER SANT HIV-1 RNA 3.0 Assay, [bDNA], Bayer Diagnostics, Berkeley, CA, USA). Results were reported in a range from 50 to >500,000 copies/ml. CD4+ count and percentage was obtained by standard flow cytometric methods.

### Statistical analysis

The software package SAS version 9.2 was used to perform the statistical analysis (Statistical Analysis System, SAS Institute Inc., Cary, NC, USA). Descriptive statistics for the continuous variables were reported as mean ± standard deviation. Categorical variables were reported as frequencies and percentages and the Chi-square test was used to test for statistical significance. The statistical level of significance was set at p < 0.05.

### Ethics

This study was conducted in accordance with the Helsinki Declaration and approved by the Research Ethics Committee and Research Advisory Council (ORA # 2031 031). Consent was waived by the KFSHRC IRB as the study was a retrospective chart review study.

## Results

### Patient Characteristics

Of the 602 patients in the cohort, 420 (70%) were male. The mean age at diagnosis was 30 years (standard deviation (SD) =14, range=1–91). KFSHRC is located within the metropolitan area of Riyadh (the country’s capital) and serves as a referral hospital for the central region of KSA. Almost one-third of the patients in the cohort were originally from this part of the country (33%, n=196). The origin of the remaining patients was broadly diversified: southern region (27%, n=161); western region (22%, n=131); eastern region (16%, n=94); and northern region (3%, n=19). During the observation period, 147 (24%) patients died and 83 (14%) were lost to follow up (Table 1) with an average of 3 patients lost to follow up per year with a peak loss of 12 patients in 2002. However, after 2010 most of the patients (62%, n=372) were still being seen for follow-up at the infectious disease clinic at KFSHRC.

A number of patients diagnosed with HIV also had HIV positive family members including spouses (14%, n=84), parents (6%, n=37), or children (5%, n=28). The major route of transmission was heterosexual contact (55%, n=329) followed by blood transfusion (24%, n=146). The paediatric age group (<14 years of age) consisted of 93 (15%) patients. Perinatal transmission was identified as the route of transmission in 59 (63%) children.

**Figure 1:**
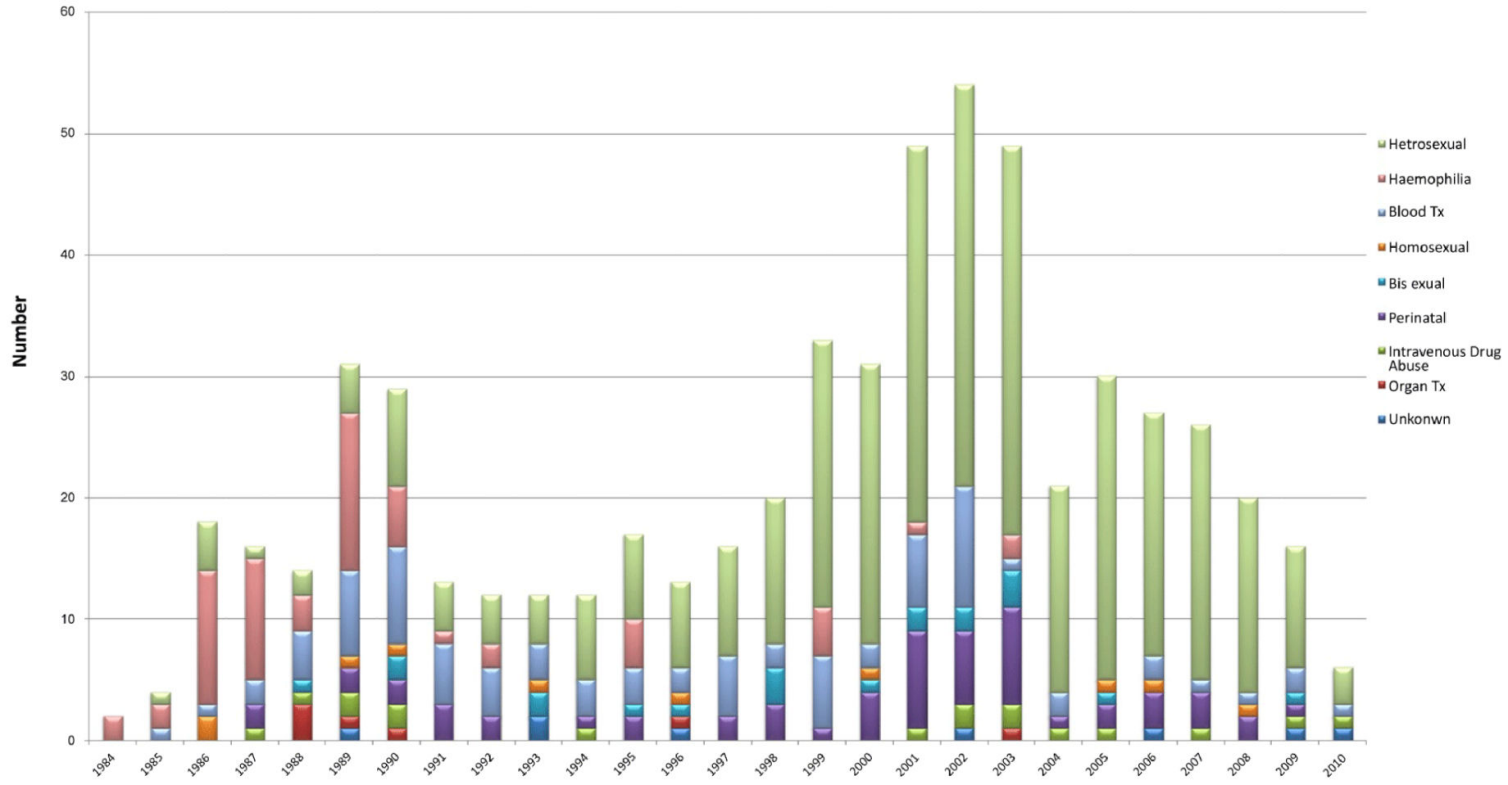
Incidence of new HIV-1 infection per year, stratified by risk group Numbers of new HIV-1 infections reported between 1984 and 2010, and stratified by mode of transmission as collected through chart review.

The annual frequency of new HIV-1 infections initially peaked between 1986 and 1990 (Figure 1). Most HIV-1 infections observed during this time were causally linked to transfusions with blood and blood products (Table 2). One patient was infected during bone marrow transplantation. Six patients acquired HIV-1 infection after kidney transplantation; two of these kidney transplants were performed as cadaveric kidneys in the United States in 1985 and four patients received the kidneys from commercial non-related donors in India and Egypt. The second peak in new HIV-1 infection occurred from 1999 to 2003 and was predominantly referrals from other hospitals within KSA. Most of these infections were linked to heterosexual transmission (65%, n=141). Since 2004, there has been a slow but steady decline in new HIV-1 patients referred from other institutions as new HIV/AIDS treatment centres have been opened around the country.

### Opportunistic infections and AIDS associated malignancies

In the 1990’s, we recorded 184 episodes of opportunistic infections and 23 AIDS associated malignancies, resulting in an OI rate of 10.6 cases per 100 patient years. Pneumocystis jirovecii pneumonia (PCP) was the most frequently diagnosed opportunistic disease (27%, n=50), followed by Candida esophagitis (25%, n=45). Mycobacterium tuberculosis (MTB) and cytomegalovirus (CMV) infections was recorded in 29 patients each and represented 16% of all opportunistic infections. Most cases of CMV infection involved retinitis (72%, n=21), pneumonitis (14%, n=4), colitis (7%, n=2), hepatitis (7%, n=2). Of the MTB infections (n=29), 59% represented pulmonary TB, 24% with disseminated infections, 10% involved the central nervous system (CNS) and 7% other sites. Atypical mycobacterial infections were diagnosed in 17 patients (9% of opportunistic infection). M. avium-intracellulare (71%), M. kansasii (12%), and M. chelonae (6%) were the most frequently isolated species. Other reported opportunistic diseases included cryptosporidiosis (n=7, 4% of opportunistic infections, four cases with intestinal disease and three cases with gastric disease), and cryptococcal infection (n=6, 3% of opportunistic infections, four cases with disseminated disease and two cases of meningitis).

Reports of AIDS associated malignancies including Kaposi sarcomas were less frequent reported and accounted for 3% in our cohort of patients (1.5% skin, 0.5% pulmonary, 0.7% gastro-intestinal, and 0.3% disseminated). Lymphoma was reported in approximately 1% of patients, 0.2% Hodgkin’s disease and 0.7% Non-Hodgkin’s Lymphoma). The mortality rate for associated malignancies is listed in Table 3.

### Treatment characteristics

**Figure 2:**
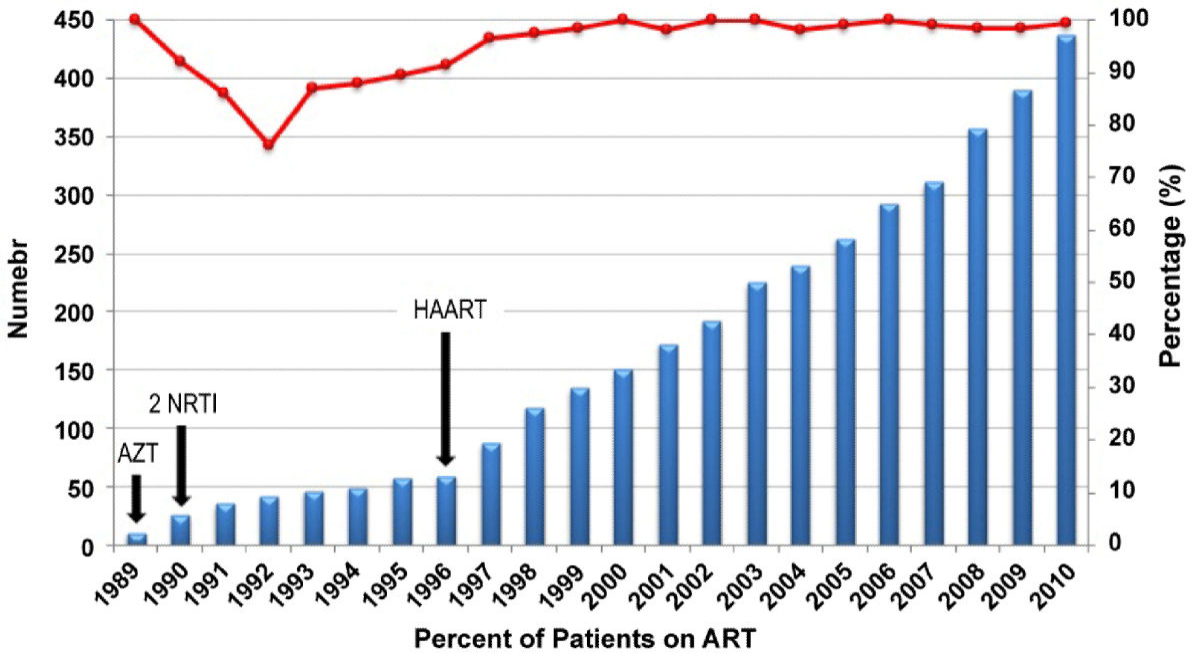
Proportion and absolute frequency of HIV-1 patients treated with antiretroviral agents (ART) The absolute number of patients are plotted by year with percentage of patients on ART treatment represented by line chart. Zidovudine (AZT) monotherapy began in 1989 (n=20). Between 1990 and 1996, two NRTI combination was used (n=56). Beginning in 1996, a combination of three or four different antiretroviral drugs (HAART) was administered.

**Figure 3:**
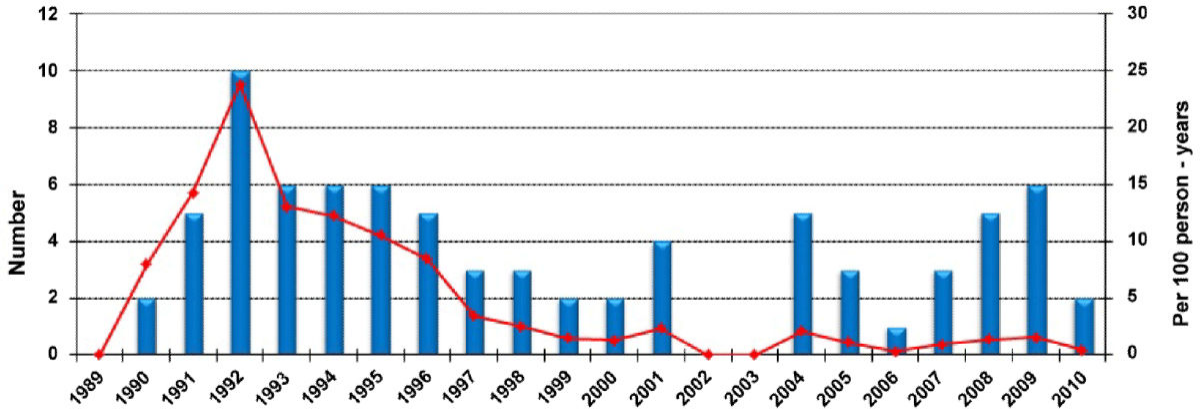
Absolute and person-year death rate from time of diagnosis Absolute number of HIV positive patient deaths per year (bar chart) compared to the death per 100 person-years per year (line chart).

Antiretroviral drugs have been available in KSA since 1987. They are used according to international standards and guidelines, for the treatment of adult and paediatric patients. Overall, the number of patients receiving antiretroviral therapy rose continuously for this cohort over the observation period (Figure 2). Zidovudine (AZT) mono-therapy was started in 1989 and administered to 20 patients. Subsequently, combination treatments with two nucleoside reverse transcriptase inhibitors (NRTI) were provided to a total of 56 patients between 1990 and 1996. Highly active antiretroviral therapy (HAART) began in 1996 as a combination of three or four antiretroviral drugs. Clinically, the initiation of HAART had dramatic impact in the mortality rate of HIV infected individuals in KSA. In our cohort, the mean CD4 count at the time of diagnosis is 350 cells/mm^3^ (performed on 244 patients at diagnosis) with an average viral load of 183, 879 copies per ml (available on 134 patients at diagnosis). Since 1996, the number of patients presenting with CD4 <200 has fallen (data not shown), and more importantly, mortality declined to 1% (Figure 3).

### Mortality

The mortality in this patient cohort was highest in the period between 1989 and 1993 peaking at 90 deaths per 1000 person-years in 1992 (Figure 3). Mortality in this cohort decreased from 90 to 20 deaths per 1000 person-years between 1993 and 2000, respectively. From 2001 to 2010 mortality declined remaining between 0 to 20 deaths per 1000 person-years, but remains significantly higher when compared to the United States rate of 0.025 deaths per 1000 person-years (http://www.cdc.gov/nchs). In addition, despite increased survival after initiation of HAART, the mortality rate of our HIV cohort at 1% remains substantially higher than the average general KSA population mortality rate 0.3% as reported by the MOH (http://data.worldbank.org).

## Discussion

According to the latest MOH report there has been a 0.5-2.5% increase in the incidence of HIV infection between 1984 and 2009 [17]. During this period, 15,213 HIV-infected persons were diagnosed with a breakdown of 4,019 Saudi nationals and 11,194 non-Saudi citizens (Annual AIDS Report-National AIDS Program www.moh.gov.sa). Given that KFSHRC being a main referral centres for HIV infection in KSA, the patient population reflected in this cohort does represent from many of the diverse geographic regions in KSA. Despite the diversity of patients, we recognize the limitation of a single centre cohort. In addition, longitudinal data was not complete for all patients, particularly those patients who may have transferred care to another facility. Even with these limitations, the longitudinal data presented for this cohort are robust and reliable given the standardization of laboratory testing, therapy regimens, and close monitoring and follow up. To our knowledge, this report is the first observational study to describe the outcome of HIV care and related mortality among a cohort of Saudi national HIV-infected patients receiving their care in KSA [14].

The modes of HIV transmission in this KSA cohort are distinct from reports in other MENA countries. In addition, we note that transmission mode followed a biphasic distribution in earlier years as compared to the recent periods. Overall, blood transfusions and blood products account for the highest fraction of HIV transmission, approximately 54%, followed by heterosexual activity at 30%. From 1984-1996, the major mode of HIV transmission was through transfusion of blood and blood products and likely reflects the lack of appropriate blood supply testing during this period. In 1996, the KSA and other GCC states established the Gulf Committee for blood banks that now follows international screening guidelines for blood banking with a subsequent drop in HIV transmission from blood products. From 1994-2010, heterosexual activity remains the dominant risk factor for HIV transmission in our cohort. Compared to other MENA countries, this result is a striking difference where intravenous drug use (IVDU) and men who have sex with men (MSM) are major modes of transmission [18,19]. However, while acquisition of HIV through homosexual activity represents a minor fraction of HIV (1.7%) transmission in our KSA cohort, this finding may also reflect reporting biases from local perceptions and stigma related to homosexuality [20]. Our data also indicate that there has been a steady increase in numbers of HIV infections acquired through IVDU with a peak in 2003 (3%) that subsequently fell. Interestingly, the rise in 2003 IVDU-related HIV acquisition increase may reflect the initiation of a screening program for HIV in the IVDU population presenting for substance abuse treatment in KSA (MOH report for UNAIDS on HIV/AIDS, 2010). With that said, there are limitations in HIV testing that could influences our cohort including the timing of HIV diagnosis, which may reflect differences in both HIV incidence and testing patterns.

In the early 1990s, the rate of opportunistic infections in our cohort is 49.7 episodes per 100 patient-years. These rates are higher than reported from developed countries at the same time period where the average opportunistic rate is 10.6 per 100 patient years [21-29]. In fact, our KSA cohort opportunistic infection rate more closely resembles those from developing countries [30-34] likely reflecting a large proportion of patients presenting with AIDS at the time of initial evaluation. On the other hand, AIDS associated malignancies are at a rate of 6 per 100 patient years, which is significantly lower compared to other studies [35] but may be due to patient attrition to medical facilities in their home regions.

The initiation of antiretroviral therapy since 1996 has been encouraging, with a general reduction of CD4+ T lymphocytes < 200 and decrease in mortality rate in this cohort. This low rate is a clear programmatic improvement in HIV treatment and intervention. In concert with the findings of CD4+ T cell preservation.- for the first time in the MENA region - we identified mortality at <1% in the latter part of the cohort between 1993 and 2010. We believe the free access to healthcare, education and the availability of antiviral therapy accounts for the maintenance of immune reconstituted HIV-infected individuals. In addition, a higher proportion of screening and treatment of infected partners contributes to this finding. However, relative to general population, mortality rates remain three times higher among HIV-infected persons (1% in HIV versus 0.3% general population). Given the growing evidence of increased cardiovascular risk, and malignancy in well-controlled HIV-infected patients, there is need for a more in depth analysis of mortality and morbidity in immune reconstituted, virally suppressed individuals in the KSA and MENA region.

## Conclusion

In conclusion, this report from a single-centre in KSA describes the dominant risk for HIV transmission from the period 1984-2010. Results confirm previous observations of delayed identification and treatment of HIV-infection, both resulting in high rates of opportunistic infections and mortality. Following initiation of antiretroviral therapy, patients experienced immune reconstitution with marked reduction in mortality. While our findings do not directly support a strategy for early case-detection of HIV, programs including a nation-wide registry and awareness campaign such as the “Get Tested” concept introduced by the CDC that recommends all individuals between the ages of 13 and 64 be tested for HIV at least once as part of routine health care - and with higher frequency if high risk factors are identified - are prudent next-steps toward improving the health status of HIV infected individuals. In addition, awareness programs and national cohort studies would help initiate early HIV treatment interventions. Based on this cohort study, future investigations into the comorbidities of well-controlled HIV infected individuals as well as those with co-infections (HBV, HCV and MTB) in the KSA and MENA region are warranted.

## Figures and Tables

**Table 1:** Socio-demographic characteristics of the HIV cohort (total of 602 patients).

Gender	Number (Percentile)	
Female	182 (30.23 %)	
Male	420 (69.76 %)	
**Age**		
Adults	507 (84.21%)	Female: 151 (29.78%) & Male: 356 (70.21%)
Pediatric	93 (15.44 %)	Female: 31 (33.33%) & Male: 64 (68.81%)
**Marital Status**		
Married	290 (48.25%)	Female: 98 (16.31%) & Male: 192 (31.95%)
Single	239 (39.77%)	Female: 37 (6.16%) & Male: 202 (33.61%)
Widowed	48 (7.99%)	Female: 37 (6.16%) & Male: 11 (1.83%)
Divorced	24 (3.99%)	Female: 9 (1.50%) & Male: 15 (2.50%)
**Partner testing**		
Positive	182 (30.23%)	Female: 101 (16.78%) & Male: 81 (13.46%)
Negative	121 (20.10%)	Female: 29 (4.82%) & Male: 92 (15.28%)
Unknown	299 (49.67%)	Female: 52 (8.64%) & Male: 247 (41.03%)
**KSA Province**		
Central Province	196 (32.56%)	Female: 54 (8.97%) & Male: 142 (23.59%)
Southern Province	161 (26.74%)	Female: 59 (9.80%) & Male: 102 (16.94%)
Western Province	131 (21.76%)	Female: 33 (5.48%) & Male: 98 (16.28%)
Eastern Province	94 (15.61%)	Female: 28 (4.65%) & Male: 66 (10.61%)
Northern Province	19 (3.16%)	Female: 8 (1.33%) & Male: 11 (1.83%)
Unknown	1 (0.17%)	Female: 0 (0%) & Male: 1 (0.17%)
**Outcome**		
Alive	372 (62%)	Female: 125 (20.76%) & Male: 247 (41.03%)
Death	147 (24%)	Female: 38 (6.31%) & Male: 109 (18.11%)
Lost follow up	83 (14%)	Female: 19 (3.16%) & Male: 64 (10.63%)

**Table 2:** Mode of HIV transmission

Transmission	Number (Percentile)	
Hemophilia receiving blood products	60 (9.97%)	Female: 0 (0%) & Male: 60 (9.97%)
Blood Transfusion	86 (14.29%)	Female: 43 (7.14%) & Male: 43 (7.14%)
Heterosexual	329 (54.65%)	Female: 102 (16.94%) & Male: 227 (37.71%)
MSM	10 (1.66%)	
Bisexual	20 (3.32%)	Female: 0 (0%) & Male: 20 (3.32%)
Perinatal	59 (9.80%)	Female: 31 (5.15%) & Male: 28 (4.65%)
Intravenous Drug Use (IVDU)	17 (2.82%)	Female: 0 (0%) & Male: 17 (2.82%)
Organ Transplantation	7 (1.16%)	
Unknown	53 (10.1%)	Female: 5 (0.83%) & Male: 48 (7.97%)

**Table 3:** Mortality rate for opportunistic infections and HIV-associated malignancies

Opportunistic Infections	Total Number of Cases	Number of Death	Percentile (%)
Pneumocystis jirovecii	50	4	8
Candida esophagitis	45	Not recorded	Not recorded
Tuberculosis (MTB)	29	9	31
Cytomegalovirus (CMV)	29	13	44.8
Cryptosporidiosis	7	4	57
Cryptococcal	6	3	50
Associated Malignancy			
Kaposi sarcomas	18	3	17
Lymphoma	6	0	0

## Abbreviations

CDC: Centers for Disease Control and Prevention
HAART: Highly Active Antiviral Therapy
IVDU: Intravenous Drug Users
MENA: **Middle East** and **North Africa** (refers to the following countries: Bahrain, Egypt, Iran, Iraq, Palestine, Jordan, Kuwait, Lebanon, Libya, Morocco, Oman, Qatar, KSA, Syria, Tunisia, UAE, and Yemen)
KSA: Kingdom of Saudi Arabia
PCR: Polymerase Chain Reaction
